# Multi-Features-Based Automated Breast Tumor Diagnosis Using Ultrasound Image and Support Vector Machine

**DOI:** 10.1155/2021/9980326

**Published:** 2021-05-19

**Authors:** Zhemin Zhuang, Zengbiao Yang, Shuxin Zhuang, Alex Noel Joseph Raj, Ye Yuan, Ruban Nersisson

**Affiliations:** ^1^Department of Electronic Engineering, Shantou University, Shantou 515063, China; ^2^School of Electrical Engineering, Vellore Institute of Technology, Vellore 632014, India

## Abstract

Breast ultrasound examination is a routine, fast, and safe method for clinical diagnosis of breast tumors. In this paper, a classification method based on multi-features and support vector machines was proposed for breast tumor diagnosis. Multi-features are composed of characteristic features and deep learning features of breast tumor images. Initially, an improved level set algorithm was used to segment the lesion in breast ultrasound images, which provided an accurate calculation of characteristic features, such as orientation, edge indistinctness, characteristics of posterior shadowing region, and shape complexity. Simultaneously, we used transfer learning to construct a pretrained model as a feature extractor to extract the deep learning features of breast ultrasound images. Finally, the multi-features were fused and fed to support vector machine for the further classification of breast ultrasound images. The proposed model, when tested on unknown samples, provided a classification accuracy of 92.5% for cancerous and noncancerous tumors.

## 1. Introduction

International Agency for Research on Cancer (IARC) reported that breast cancer accounts for about 24.2% of cancers diagnosed in women worldwide [1]. It is also the leading fatal cause in women, accounting for about 15%. With the development of modern medicine, if breast cancer is diagnosed early, the survival rate of patients will be significantly improved [2]. Breast tumors are usually examined by computerized tomography (CT), magnetic resonance imaging (MRI), molybdenum target X-ray, far infrared, ultrasound (US), and other methods. Among them, the US has become the preferred choice for early breast cancer screening due to its cost-effectiveness and more robust images [3]. However, the Breast Imaging-Reporting and Data System (BI-RADS) [4] grades diagnosed by different clinicians for the same patient are subjective and different since some features in the breast ultrasound (BUS) images are not typically visible to diagnose [5]. Besides, different breast lesions show different features in BUS images. Also, experience and the ability to understand the visual clues from BUS images are essential in reducing false negative detection. The count shows that the missed diagnosis of medical imaging in disease diagnosis can be between 10% and 30% [6].

Artificial intelligence (AI) can assist doctors in making a more accurate judgment because of its objectivity and versatility. AI diagnosis of benign and malignant BUS images can be divided into deep learning and feature extraction. Deep learning transforms the raw BUS images into much higher-dimension expression through convolutional neural networks (CNNs). Multi-level and multi-aspect features are obtained by training the network model, which makes the obtained features have a more robust generalization and expression. Deep learning is often used in the automatic classification of BUS images. For example, in reference [7], 166 malignant and 292 benign BUS images were trained and classified by using a neural network composed of three convolution layers and two fully connected layers. Qi et al. [3] used Mt-Net (malignant tumors) and Sn-Net (solid nodules) to classify BUS images, where Mt-Net was used to detect malignant tumors and Sn-Net was used to detect solid nodules. Although deep learning has achieved good results, they are constrained by the need for a higher number of ground truth (GT).

Feature extraction techniques identify useful characteristic features (CFs) from the original images, where the original image is transformed into a group of features with obvious physical significance, to achieve the purpose of dimensionality reduction. For example, in reference [8], the region growing method was used to segment the lesion, and the histogram method was used to calculate six histograms from the posterior shadowing region (PS). Finally, BUS images were divided into PS enhancement and PS nonenhancement by using the six histograms and multilayer perceptron (MLP). However, PS is only one of the features to judge the benign and malignant BUS images and lacks accuracy to make a classification.

In this paper, (1) by analyzing the different manifestations of benign and malignant breast tumors in ultrasound images, combined with the clinical experience of experts, different and effective characteristic features were designed manually. (2) In order to assist the classification of breast tumor ultrasound images, we used transfer learning for extracting deep learning features. Finally, (3) SVM was used to integrate characteristic features and deep learning features and present an effective classification.

## 2. Materials and Methods

**Figure 1:**
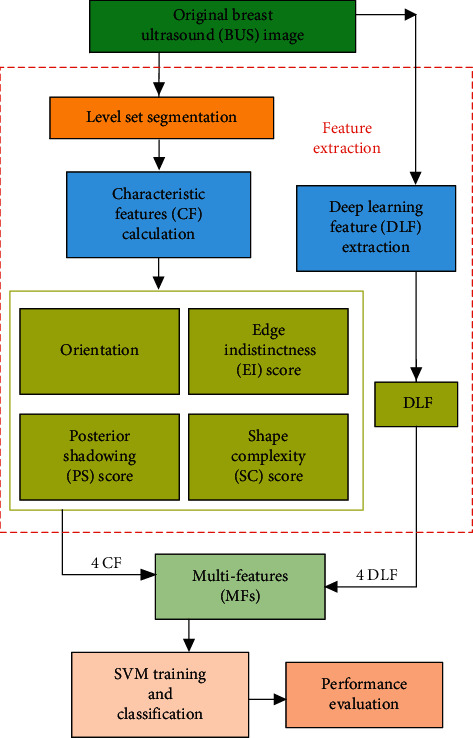
The proposed methodology.

Since benign and malignant breast BUS images have different histological structures and biological characteristics, they exhibit different properties on BUS images. The malignant tumors such as ductal carcinoma in situ [9], due to their invasive nature, penetrate through the ducts and into adjacent fibrous and adipose tissues. It forms a blurred mixed zone between the tumor and the tissues and complex edge. Besides, the complex interstitial components and hyaline transformation of malignant lesions often lead to scattering of acoustic signals [10, 11]. During the decision process, the specialists often consider the orientation, posterior shadowing (PS), edge indistinctness (EI), and shape complexity (SC) of the tumors as essential features to identify them as benign or malignant. These characteristic features (CFs) of BUS images are not only a critical judgment for clinical diagnosis but also a significant basis for BUS image classification based on feature extraction. Hence, we proposed a method to classify breast lesions using multi-features (MFs) and SVM. The proposed method firstly employed a level set technique to segment the tumor region of BUS images. From the contour of the segmented tumor, orientation and edge indistinctness (EI) scores were calculated. Next, using Hu moments, we determined the characteristic of the posterior shadowing (PS) region. Later, the fractal divider method was used to calculate the shape complexity (SC) score of tumor contour. Meanwhile, the pretrained VGG16 model was used as a feature extractor to get deep learning features (DLFs) of BUS images. Finally, we classified the BUS images based on the multi-features (MFs) obtained above and SVM. The above process is illustrated in Figure 1.

## 3. Lesion Segementation and Feature Calculation

Due to the complexity involved during the ultrasound examination, the acquired images contain speckle noise, image artifacts, and weak boundaries that hurt the segmentation process. Accurate segmentation can effectively improve the accuracy of classification [12]. Therefore, for accurate extraction of tumor regions, conventional segmentation techniques may not provide desired results. Literature suggests that level set techniques are useful for segmentation problems related to topological changes, and hence we used an advanced level set segmentation algorithm based on geometric active contour model and curve evolution theory to complete the lesion segmentation of BUS images [13]. The technique employed an iterative method to segment the tumor region within the BUS image accurately. The following paragraph briefly explains the use of a level set algorithm to segment the BUS images.

The level set algorithm that we used in this paper does not depend on the gradient information of the BUS image. Therefore, it is insensitive to noise and has a significant advantage in medical image processing [14, 15]. Here we employed the Distance Regularized Level Set Evolution (DRLSE) [16] model that eliminates the need for reinitialization but employs a distance regularization term and energy functions to propagate the zero-level set function (LSF) towards the desired locations. The energy function *E*(*ϕ*) can be defined as follows.

For *ϕ* : Ω⟶*ℜ*,

(1) $$\begin{array}{c} E\left(\phi \right)=\mu \int_{\Omega }p\left(\left|\nabla \phi \right|\right)\text{d}x+\lambda \int_{\Omega }g\delta \left(\phi \right)\left|\nabla \phi \right|\text{d}x+\alpha \int_{\Omega }gH\left(-\phi \right)\text{d}x, \end{array}$$

where ∫_Ω_*p*(|∇*ϕ*|)d*x* is the regularization term and ∫_Ω_*gδ*(*ϕ*)|∇*ϕ*|d*x* and ∫_Ω_*gH*(−*ϕ*)d*x* are the external energy terms. *μ* > 0, *λ* > 0, *α* ∈ *ℜ* are the coefficients, respectively, *H* and *δ* are the Heaviside function and Dirac delta function, respectively, and *p* is a potential function.

Due to the addition of the distance regularization term *p*(|∇*ϕ*|) in equation (1), the deviation between the level set and the signed distance function (SDF) is automatically corrected in each iteration of the level set function, thus maintaining stability and avoiding reinitialization.

The following gradient descent flow function can be obtained by differentiating equation (1), to realize the extraction of the tumor region in BUS image while minimizing the energy function.

(2) $$\begin{array}{c} \frac{\partial \phi }{\partial t}=\mu \left[\Delta \phi -\text{div}\left(\frac{\nabla \phi }{\left|\nabla \phi \right|}\right)\right]+\lambda \delta_{\varepsilon }\left(\phi \right)\text{div}\left(g\frac{\nabla \phi }{\left|\nabla \phi \right|}\right)+\alpha g\delta_{\varepsilon }\left(\phi \right). \end{array}$$

**Figure 2:**
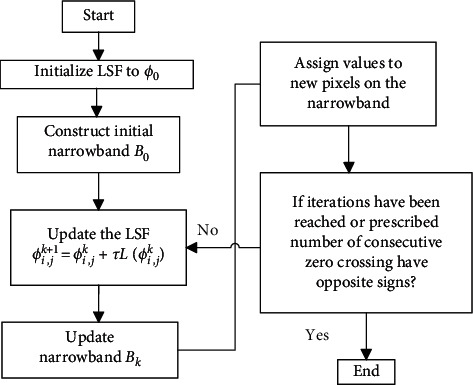
The evolution process of DRLSE.

**Figure 3:**
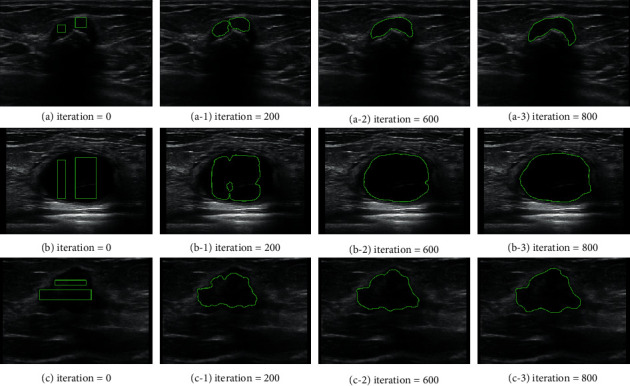
Segmentation results from the DRLSE model: (a), (b), and (c) correspond to zero LSF. (a-1) to (a-3), (b-1) to (b-3), and (c-1) to (c-3) illustrate the evolution of the LSF at 200, 600, and 800 iterations, respectively.

The implementation of DRLSE for an application is based on the flowchart illustrated in Figure 2, which includes (a) initialization of LSF *ϕ*_0_ and narrowband *B*_0_; (b) updating the LSF and narrowband region; (c) updating the pixel values on the narrowband based on *ϕ*_*i*,*j*_^*k*+1^, where (*i*, *j*) is called zero-crossing point, k is the number of iteration, and *τ* is the time step; and (d) termination of the process, if the prescribed number of consecutive zero-crossing has opposite signs or the expected number iterations is reached. The segmentation results of the lesions in breast ultrasound images are shown in Figure 3.

### 3.1. Computation of Orientation

**Figure 4:**
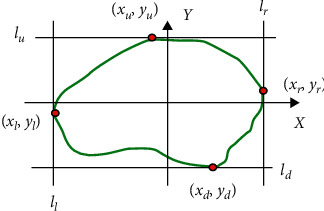
The contour of the segmented tumor region of the BUS image. (*x*_*u*_, *y*_*u*_), (*x*_*d*_, *y*_*d*_), (*x*_*l*_, *y*_*l*_), and (*x*_*r*_, *y*_*r*_)are the four vertices obtained from the intersection of lines *l*_*u*_,*l*_*d*_, *l*_*l*_, and *l*_*r*_ with the upper, lower, leftmost, and rightmost extreme of contour, respectively.

The growth characteristics of benign and malignant tumors vary and therefore show different orientations. Here we used contour obtained from the segmentation process to facilitate the calculation of tumor orientation. First, we transversed the segmented contour in both horizontal and vertical directions and obtain four points: top (*x*_*u*_, *y*_*u*_), bottom (*x*_*d*_, *y*_*d*_), leftmost (*x*_*l*_, *y*_*l*_), and rightmost (*x*_*r*_, *y*_*r*_). These points are vertices obtained from the intersection of lines *l*_*u*_, *l*_*d*_, *l*_*l*_, and *l*_*r*_ with upper, lower, leftmost, and rightmost extreme regions of contour, respectively, as shown in Figure 4.

Next, using equation (3), we computed the tumor orientation, which is expressed as a ratio between the height to the width of the tumor.

(3) $$\begin{array}{c} \text{Orientation}=\frac{y_{u}-y_{d}}{x_{r}-x_{l}}. \end{array}$$

### 3.2. Computation of Edge Indistinctness (EI) Score of Lesions

**Figure 5:**
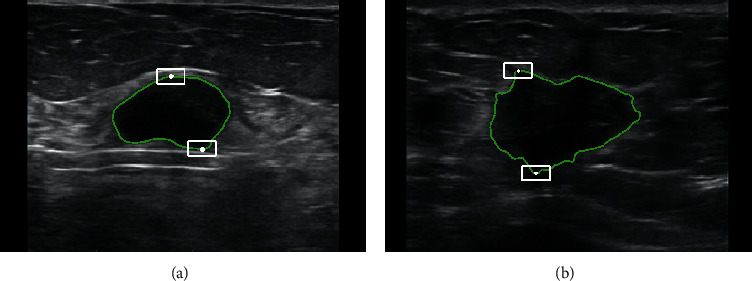
(a) and (b) illustrate segmented contour shown in green and selected *n* × *m* regions around (*x*_*u*_, *y*_*u*_) and (*x*_*d*_, *y*_*d*_) points in white, respectively. Here we set *n* = 15 and *m* = 21.

Commonly malignant tumors penetrate deeper into the tissues causing indistinctive margins that are different from the benign ones. Therefore, to measure the edge indistinctness more comprehensively, we extracted a region of *m* x *n* pixels from the top and bottom vertices of the contour, i.e., around (*x*_*u*_, *y*_*u*_) and (*x*_*d*_, *y*_*d*_) points, as shown in Figure 5.

For the extracted *m* × *n* region, we separately calculated the standard deviation along the row and the column as given in equations (4) and (5). We defined EI score (equation (6)) as the maximum of the standard deviation computed along with the row and column directions:

(4) $$\begin{array}{c} x_{\text{std}}=\frac{\sum_{i=1}^{n}x_{\text{std\_}i}}{n}, \end{array}$$

(5) $$\begin{array}{c} y_{\text{std}}=\frac{\sum_{j=1}^{m}y_{\text{std\_}j}}{m}, \end{array}$$

(6) $$\begin{array}{c} \text{EI score}=\max\left(x_{\text{std}},y_{\text{std}}\right). \end{array}$$

For our experiments, we have selected two regions around (*x*_*u*_, *y*_*u*_) and (*x*_*d*_, *y*_*d*_) points and accordingly computed two EI scores: EI score_up_ and EI score_down_, respectively.

In cancer diagnosis, the edge strength (blurring) is an important index that is used to classify the tumor. However, there are differences in the degree of blurring across different sections along the tumor boundary. Thus, we defined the average values of EI score_up_ and EI score_down_ as EI score, as shown in the following equation:

(7) $$\begin{array}{c} \text{EI score}=\frac{\left(\text{EI }{\text{score}}_{\text{up}}+\text{EI }{\text{score}}_{\text{down}}\right)}{2}. \end{array}$$

### 3.3. Computation of the PS Score of Posterior Shadowing Region by Using Hu Moments

**Figure 6:**
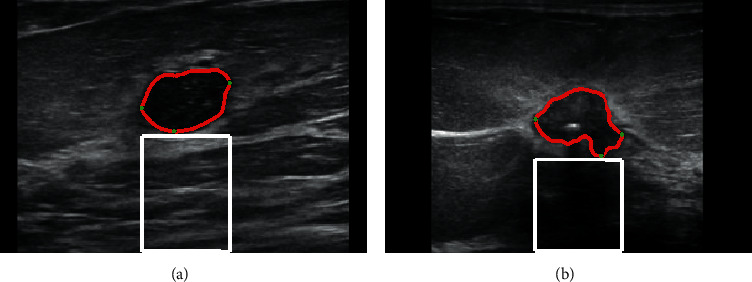
The PS regions. (a) and (b) are the benign and malignant BUS images, respectively. The red line illustrates the segmented tumor contour; the white region represents the PS region considered for PS score computation.

The texture characteristics of the PS region are generally different for benign and malignant tumors. Literature suggests that moments can be used for analyzing texture characteristics [17], and therefore, we used moment invariants proposed by Hu [18] to compare the PS regions of different BUS images quantitatively. The PS region of the BUS image was extracted based on the bottom (*x*_*d*_, *y*_*d*_), the leftmost (*x*_*l*_, *y*_*l*_), and the rightmost (*x*_*r*_, *y*_*r*_) points of the contour, as shown in Figure 6.

Let *f*(*x*, *y*) be the extracted PS region; then, its (*p*+*q*) order of the geometric moment can be defined as follows:

(8) $$\begin{array}{c} m_{pq}=\sum_{y=1}^{N}\sum_{x=1}^{M}x^{p}y^{q}f\left(x,y\right),\;p,q=0,1,2\ldots . \end{array}$$

Their central moments can be defined as

(9) $$\begin{array}{c} \mu_{pq}=\sum_{y=1}^{N}\sum_{x=1}^{M}{\left(x-\bar{x}\right)}^{p}{\left(y-\bar{y}\right)}^{q}f\left(x,y\right),\;p,q=0,1,2\ldots , \end{array}$$

where $\bar{x}$ and $\bar{y}$ are the center of gravity coordinates of the image, given by

(10) $$\begin{array}{c} \bar{x}=\frac{m_{10}}{m_{00}},\\ \bar{y}=\frac{m_{01}}{m_{00}}. \end{array}$$

The normalized central moment is defined as

(11) $$\begin{array}{c} \eta_{pq}=\frac{\mu_{pq}}{\mu_{00}^{\rho }}, \end{array}$$

where *ρ*=((*p*+*q*)/2)+1.

Normally, seven Hu moments can be computed using the second- and third-order normalized central moments. But here we only use the first moment as it is sufficient to provide a score that could differentiate PS regions of different BUS images effectively. Accordingly, we substituted *p* = 0 and *q* = 2 in equation (11) and defined the PS score as

(12) $$\begin{array}{c} \text{PS score}=\eta_{20}+\eta_{02}. \end{array}$$

To have a clear distinction, the PS score is transformed as

(13) $$\begin{array}{c} \text{PS score}=-\text{sign}\left(\text{PS score}\right)*\left(\text{log}10\left(abs\left(\text{PS score}\right)\right)\right). \end{array}$$

### 3.4. Computation of Shape Complexity (SC) Score Based on Fractal Dimensions

The shape is one of the critical factors clinical experts use to classify tumors as benign or malignant. Cancerous tumors have complex contours, whereas benign ones have simpler structures. Therefore, we proposed a technique based on fractals to quantify the shape complexity of the segmented tumors. In image processing, fractals have been widely used in US image segmentation. Omiotek et al. [19] used fractals to quantify the texture of thyroid US images. Lin et al. [20] used fractals to determine the area of alveolar bone defect, and recently Zhuang et al. [21] collectively used fuzzy enhancement and fractal length to segment the US image of atherosclerosis successfully. Also, the fractal theory was successfully used to measure the irregular coastlines and the fault geometries propagated by earthquakes. For example, Mandelbrot [22] employed power law to relate the costal length to the different linear rulers, and Okubo and Aki [23] quantified complex fault geometries to large values of fractal dimension.

**Figure 7:**
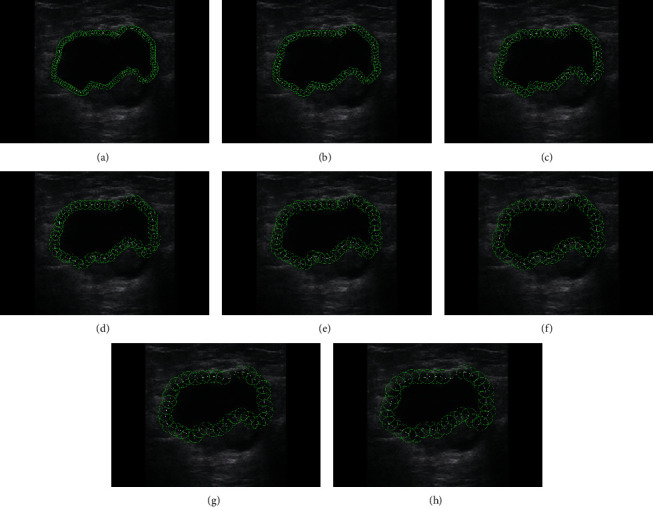
Illustration of divider method. (a)–(h) show the circles drawn with different radii along the contour of the segmentation tumor. (a) *R* = 4. (b) *R* = 5. (c) *R* = 6. (d) *R* = 7. (e) *R* = 8. (f) *R* = 9. (g) *R* = 10. (h) *R* = 11.

Here the divider method used by cartographers [23–25] was employed to quantify the shape complexity of the segmented tumor. To measure the complexity, we drew circles of different radii along the boundary of the segmented contour. The starting point was chosen as the top point (*x*_*u*_, *y*_*u*_) and the circles of different radii were drawn dividing the contours, as shown in Figure 7. Let *N* (*R*) be the number of the circles and *R* be their corresponding radius; then, according to [26, 27], we can relate *N* (*R*) and *R* as

(14) $$\begin{array}{c} {\text{Log}}_{10}N\left(R\right)=a+b{\text{Log}}_{10}R, \end{array}$$

where “*a*” and “*b*” are constants that are obtained through least-squares fit between Log_10_*N*(*R*) and Log_10_*R* and “*b*” is the slope of the line that represents the fractal dimension, which determines the SC score of the contour.

**Figure 8:**
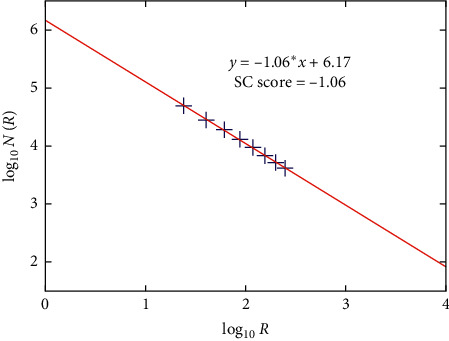
Computation of SC score for the measurements recorded in Table 1 on equation (15).

The illustration of the divider method for a sample BUS image is shown in Figure 7, and Table 1 presents *R* and their corresponding *N* (*R*) values. Further Figure 8 demonstrates the SC score obtained by the least-square fitting of *N* (*R*) and *R* for the sample BUS image shown in Figure 7.

## 4. Deep Learning Features (DLF) Extraction and SVM-Based Classification

In the above, we identified handcrafted features such as shape score, PS score, and EI and orientation to identify the characteristic features (CFs) of the segmented lesion in a BUS image. Here we retrieved high-dimension features from the BUS image to assist the classification. A deep learning method can fix this problem by performing convolution operations on the input graphics multiple times. Therefore, by using deep learning, we extracted the DLF of the image and combined them with the manually extracted CF to distinguish benign and malignant BUS images.

**Figure 9:**
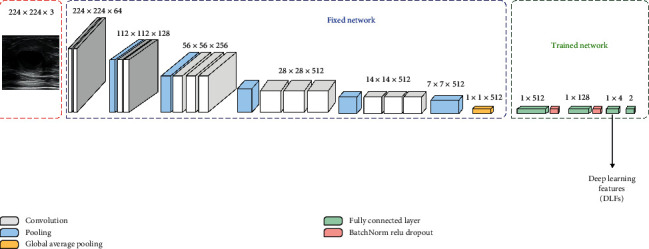
Modified VGG16.

Due to the limited dataset, our paper used transfer learning [28] to train the neural network as the DLF extractor. Initially, we compared the classification ability of VGG16 [29], VGG19 [29], ResNet50 [30], and Inception V3 [31] for BUS images. The experiment results show that VGG16 is better than other networks. Then, based on transfer learning, we froze the convolution layer of VGG16, added the global average pooling layer, and changed 7 × 7 × 512 of the original VGG16 output into 1 × 1 × 512. For the full connected layer, the nodes were set to 512, 128, 4, and 2, respectively. At the same time, the Relu activation function was added after each fully connected layer except the last one. The modified VGG16 is shown in Figure 9. For the output, the cross-entropy loss function was used to calculate the loss. After training, for other input images, we could get four values (DLF) from the second fully connected layer of the trained model.

Once the features have been computed from the BUS images, we used the SVM classifier to classify them as benign or malignant. The SVM was chosen since (a) the availability of labeled large medical image data sets for training is not feasible. SVM could provide better classification accuracy for smaller training sets [32]; (b) SVM theory provides a way to avoid inseparable problem in low-dimensional space through the use of kernel functions [33]. This attribute simplifies the problems in higher-dimensional space providing a more generalized classification.

Normally, to solve the problem of linear inseparability in low-dimensional space, kernel functions are used to map the features from low-dimensional space to high-dimensional space, thus realizing the linear classification in higher-dimensional space. In the experiments, the radial basis function (RBF) [34] was used as the kernel function of SVM to classify the CF, the DLF, and the MF, respectively, and the classification results were evaluated by various evaluation indexes.

## 5. Results

For experimental results, we have used 1802 BUS images that include 787 benign and 1015 malignant BUS images. It contains two parts. The first part is provided by Ultrasoundcases.info, which is a professional breast cancer ultrasound website developed by Hitachi Medical Systems in Switzerland and Dr. Taco Geertsma, who works for Gelderse Vallei hospital in the Netherlands. It contains many ultrasound cases, which were collected and labeled by radiologists and ultrasound technicians of the hospital over the years. The other part is supported by the First Affiliated Hospital of Shantou University, Guangdong Province, China.

### 5.1. Evaluation Indexes

The following measures (equations (15)–(19)) were used as a metric to evaluate the performance of the SVM classifier model [35].

(15) $$\begin{array}{c} \text{Sensitivity}=\frac{\text{TP}}{\text{TP}+\text{FN}}, \end{array}$$

(16) $$\begin{array}{c} \text{specificity}=\frac{\text{TN}}{\text{TN}+\text{FP}}, \end{array}$$

(17) $$\begin{array}{c} \text{accuracy}=\frac{\text{TP}+\text{TN}}{\text{TP}+\text{TN}+\text{FP}+\text{FN}}, \end{array}$$

(18) $$\begin{array}{c} F1-\text{score}=\frac{2\text{TP}}{2\text{TP}+\text{FP}+\text{FN}}, \end{array}$$

(19) $$\begin{array}{c} \text{precision}=\frac{\text{TP}}{\text{TP}+\text{FP}}, \end{array}$$

where true positive (TP): GT malignant and prediction malignant; false positive (FP): GT benign and prediction malignant; false negative (FN): GT malignant and prediction benign; and true negative (TN): GT benign and prediction benign.

### 5.2. Characteristic Feature (CF) Calculation

**Figure 10:**
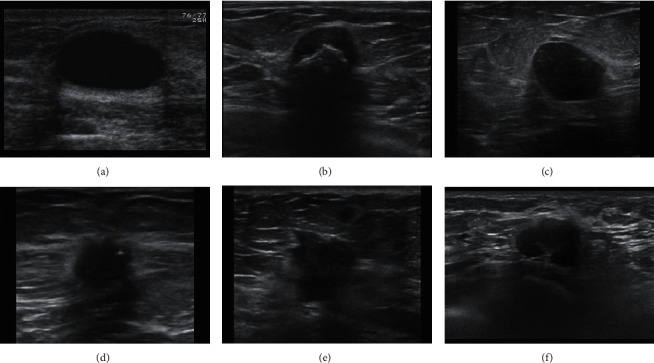
BUS images. (a), (b), and (c) are benign. (d), (e), and (f) are malignant.

To illustrate the calculation of CF, we presented 6 BUS images as examples, as shown in Figure 10. The calculation results of CF are listed in Table 2. The original value is calculated by using the proposed methods, and the normalized value presents the normalized CF value.

It can be seen from Table 2 that the characteristic features (CFs) of Figures 10(a) and 10(d) are consistent with the biological properties of benign and malignant BUS images. For example, Figure 10(a) which was diagnosed as benign tumor has larger EI score compared with malignant tumor (Figure 10(d)). However, we can also find that benign and malignant BUS images may have the same properties, for example, the posterior shadowing (PS) shown in Figures 10(b) and 10(f) is relatively low. Therefore, only relying on a single feature to distinguish benign and malignant BUS images will present a higher probability of misjudgment.

### 5.3. Deep Learning Feature (DLF) Extractor

To select the best DLF extractor, based on transfer learning, we used VGG16, VGG19, ResNet50, and Inception V3 to train and test the dataset, which is composed of 955 malignant and 727 benign BUS images. In the experiment, the training set accounts for 80% of the total data and the remaining accounts for the test set. The experimental results are shown in Table 3.

As we can see from Table 3, the accuracy, sensitivity, specificity, and *F*1-score of VGG16 are 0.84, 0.86, 0.82, and 0.86, respectively, which are higher than those of VGG19, ResNet50, and Inception V3. From the table, it can be inferred that VGG16 has better classification ability; it can learn BUS image features better than other networks. Therefore, we use VGG16 to extract deep learning features (DLFs) to assist the SVM classification.

### 5.4. Classification of Breast Tumors Based on SVM

In this experiment, we used another 120 BUS images, which are totally different from the data used in the above section. Among them, 80 BUS images were used to train the SVM model, including 40 benign BUS images and 40 malignant BUS images. Also, we took another 40 BUS images as the test set. For those 120 BUS images, we extracted their characteristic features (CFs) and deep learning features (DLFs) and then concatenated them serially to form multi-features (MF), as shown in equation (20). Later, the MF was normalized and labeled for supervised learning. We use “1” to indicate malignant and “0” to label benign. After the preparation, we firstly used triple cross-validation on the training samples to get the best classifier and then used the classifier to test the training samples and test samples, respectively.

(20) $$\begin{array}{c} \text{MF}=\left\{\text{CF},\text{DLF}\right\}, \end{array}$$

where,

(21) $$\begin{array}{c} \text{CF}=\left\{\text{orientation},\text{EI score},\text{PS score},\text{SC score}\right\}. \end{array}$$

**Figure 11:**
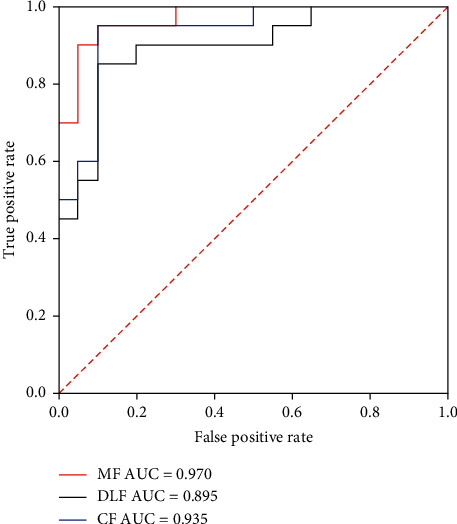
ROC curve and AUC value by using characteristic features (CFs), deep learning features (DLFs), and multi-features (MFs), respectively.

In Table 4, CF and a different number of deep learning features (DLFs) are used as multi-features (MFs) to classify BUS images based on SVM. The result shows that the MF composed of CF and 4 DLF could get better classification results. Therefore, we choose CF and 4 DLF as the final MF. Further, from Table 5, it can be seen that by using SVM, the classification accuracy, precision, sensitivity, specificity, and *F*1-score of MF are 0.925, 0.905, 0.95, 0.905, and 0.927, respectively, which are higher than those indicators obtained from other classification methods. In Figure 11, the ROC curve and AUC value of SVM classification based on characteristic features (CFs), four deep learning features (DLFs), and multi-features (MFs) are recorded. The results show that the AUC value of MF is 0.970, which is higher than that of CF (0.935) or 4 DLF (0.895). Therefore, the classification model based on MF is better than other classification techniques.

### 5.5. Triple Cross-Validation

Here, we used triple cross-validation to illustrate the advantages of using the multi-features (MF), that is, for different *c*, *g* values in SVM, the training dataset is randomly divided into three parts: two of them are considered as the training set and the rest is used for verification set. The average accuracy of the three validation sets is considered as the accuracy of the SVM with these *c*, *g* values. Here *c* = 0.5 is the regularization parameter of SVM, and *g* = 0.25 is the parameter of radial basis function (RBF).

**Figure 12:**
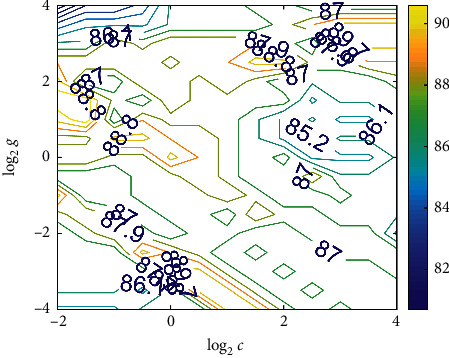
Triple cross-validation of multi-features (MFs).

The contour plot in Figure 12 represents different accuracy values obtained when different *c* and *g* were used during triple cross-validation. After the triple cross-validation, the *c*, *g* values that represent the highest accuracy among the triple cross-validation were considered as the parameters of the final SVM classifier. The accuracy among the triple cross-validation can reach over 88%, which is close to the final accuracy of 92.5% obtained by using the best *c*, g. This indeed illustrates that the model has good robustness.

## 6. Discussion

Recently, various models relied on deep learning that has been used in BUS image classification. For example, references [36, 38–40] used deep learning methods to extract BUS image features and present classification. Compared with the direct use of traditional deep learning models such as VGG16, VGG19, ResNet50, and Inception V3, they got better results. However, they have the following problems. First of all, because CAD system is mainly used to assist doctors in BI-RADS classification of breast tumors, rather than just relying on CAD system to determine benign and malignant breast tumors, the quantitative methods we proposed can better assist doctors in diagnosis. In addition, according to the clinical experience of doctors, on the basis of segmentation, we quantify the regions with different characteristics of benign and malignant breast tumors, such as posterior shadowing (PS), edge indistinctness (EI), and so on, so as to avoid the influence of irrelevant areas in BUS images on the experimental results and reduce the interference of inherent noise of BUS images.

## 7. Conclusions

In this work, firstly, with the help of the clinical experience of doctors, four characteristic features (CFs) of BUS images were obtained manually, including orientation, edge indistinctness (EI), characteristics of posterior shadowing region (PS), and shape complexity (SC). Based on the experiments, we compared the CF computed from different BUS images, which showed that the CF designed in this paper can characterize the different properties of benign and malignant BUS images. Meanwhile, the experiment showed that using a single feature to distinguish BUS images was prone to false interpretation. Also, this paper introduced deep learning features (DLFs) to improve the accuracy of classification further. In the experiment of DLF extraction, through comparing the classification results of several classical deep neural networks, it was found that the accuracy, sensitivity, specificity, and *F*1-score of VGG16 are 0.84, 0.86, 0.82, and 0.86, respectively, which were higher than those of other classical neural networks. Therefore, we employed a modified VGG16 as a deep learning feature extractor followed by SVM to classify the fused the CF and DLF. The results showed that the accuracy, sensitivity, specificity, and *F*1-score of this method are 92.5%, 90.5%, 95%, 90.5%, and 92.7% respectively, which were better than other methods. Finally, through the triple cross-validation of multi-features (MFs), the experiment results further indicated that the proposed method can be used to assist doctors to identify benign and malignant BUS images effectively.

## Figures and Tables

**Table 1:** Number of circles *N* (*R*) and their radius *R* for BUS image shown in Figure 7.

Figure 7	(a)	(b)	(c)	(d)	(e)	(f)	(g)	(h)
*R*	4	5	6	7	8	9	10	11
*N* (*R*)	109	85	72	61	53	46	43	37

**Table 2:** Calculation results of CF in Figure 10 (Figures 10(a)–10(c) are benign and Figures 10(d)–10(f) are malignant) (EI—edge indistinctness, PS—posterior shadowing, SC—shape complexity, OV—original value, and NV—normalized value).

Images	Orientation		EI		PS score		SC	
	OV	NV	OV	NV	OV	NV	OV	NV
(a)	0.544	0	27.530	0.572	2.751	1	−1.021	0
(b)	0.670	0.283	27.529	0.572	1.983	0	−1.025	0.05
(c)	0.835	0.654	35.151	1	2.248	0.371	−1.027	0.008
(d)	0.878	0.751	17.329	0	2.321	0.440	−1.077	0.7
(e)	0.989	1	30.194	0.722	2.474	0.693	−1.101	1
(f)	0.772	0.512	23.215	0.33	2.153	0.221	−1.034	0.163

**Table 3:** The classification result of using classical deep neural network.

Pretrained models	Accuracy	Sensitivity	Specificity	*F*1-score
VGG16	0.84	0.86	0.82	0.86
VGG19	0.81	0.81	0.77	0.82
ResNet50	0.78	0.77	0.77	0.85
InceptionV3	0.78	0.83	0.7	0.81

**Table 4:** The comparison of classification using different numbers of DLF and CF (CFs—characteristic features; DLFs—deep learning features).

Classification models	Accuracy	Sensitivity	Specificity	*F*1-score
2 DLF + CF + SVM	0.875	0.895	0.85	0.872
4 DLF + CF + SVM	0.925	0.905	0.95	0.927
6 DLF + CF + SVM	0.9	0.9	0.9	0.9
8 DLF + CF + SVM	0.85	0.889	0.8	0.84
10 DLF + CF + SVM	0.875	0.941	0.8	0.84
12 DLF + CF + SVM	0.85	0.889	0.8	0.84
16 DLF + CF + SVM	0.875	0.895	0.85	0.872

**Table 5:** The comparison of classification methods (CFs—characteristic features, DLFs—deep learning features, and MFs—multi-features).

Models	Accuracy	Precision	Sensitivity	Specificity	*F*1-score
MF + SVM	**0.925**	**0.905**	**0.95**	**0.905**	**0.927**
CF + SVM	0.875	0.895	0.85	0.895	0.827
DLF + SVM	0.8	0.875	0.7	0.875	0.778
[3]	0.901	NA	0.935	0.832	NA
[7]	0.83	NA	NA	0.824	NA
Inception V3 [36]	0.78	NA	0.77	0.78	NA
VGG19 [36]	0.82	0.70	0.70	0.78	NA
[37]	0.8667	NA	0.9245	0.7838	NA

The bold values indicate that the result of the proposed method is better than that of other classification methods.

## Data Availability

Part of the datasets used in this study can be found on the following website: https://www.ultrasoundcases.info/cases/breast-and-axilla/, and the other part is the data provided by the hospital, which is not open to the public because it involves research privacy.
